# The Fluorescent D-Amino Acid NADA as a Tool to Study the Conditional Activity of Transpeptidases in *Escherichia coli*

**DOI:** 10.3389/fmicb.2018.02101

**Published:** 2018-09-04

**Authors:** Alejandro Montón Silva, Christian Otten, Jacob Biboy, Eefjan Breukink, Michael VanNieuwenhze, Waldemar Vollmer, Tanneke den Blaauwen

**Affiliations:** ^1^Bacterial Cell Biology and Physiology, Swammerdam Institute for Life Sciences, University of Amsterdam, Amsterdam, Netherlands; ^2^Centre for Bacterial Cell Biology, Institute for Cell and Molecular Biosciences, Newcastle University, Newcastle upon Tyne, United Kingdom; ^3^Department of Membrane Biochemistry and Biophysics, Institute of Biomembranes, Utrecht University, Utrecht, Netherlands; ^4^Department of Chemistry, Indiana University Bloomington, Bloomington, IN, United States

**Keywords:** *E. coli*, LdtD, peptidoglycan, transpeptidases, NADA, aztreonam, cell division

## Abstract

The enzymes responsible for the synthesis of the peptidoglycan (PG) layer constitute a fundamental target for a large group of antibiotics. The family of β-lactam antibiotics inhibits the DD-transpeptidase (TPase) activity of the penicillin binding proteins (PBPs), whereas its subgroup of carbapenems can also block the TPase activity of the LD-TPases. D-Ala fluorescent probes, such as NADA, are incorporated into the PG presumably by TPases in *Escherichia coli* and can be used to study conditions that are required for their function. Of all LD-TPases of *E. coli*, only LdtD was able to incorporate NADA during exponential growth. Overproduction of LdtD caused NADA to be especially inserted at mid cell in the presence of LpoB-activated PBP1b and the class C PBP5. Using the NADA assay, we could confirm that LpoB activates PBP1b at mid cell and that CpoB regulates the activity of PBP1b *in vivo*. Overproduction of LdtD was able to partly compensate for the inhibition of the cell division specific class B PBP3 by aztreonam. We showed that class A PBP1c and the class C PBP6b cooperated with LdtD for NADA incorporation when PBP1b and PBP5 were absent, respectively. Besides, we proved that LdtD is active at pH 7.0 whereas LdtE and LdtF are more active in cells growing at pH 5.0 and they seem to cooperate synergistically. The NADA assay proved to be a useful tool for the analysis of the *in vivo* activities of the proteins involved in PG synthesis and our results provide additional evidence that the LD-TPases are involved in PG maintenance at different conditions.

## Introduction

Peptidoglycan (PG) maintains the shape of bacterial cells and protects them against bursting due to the osmotic pressure. In Gram-negative bacteria, the PG layer is sandwiched between the cytoplasmic and outer membrane in the periplasm (Vollmer et al., 2008a). PG is linked to the outer membrane by covalent linkage with Braun’s lipoprotein and by non-covalent interactions with proteins such as OmpA (Magnet et al., 2008; Samsudin et al., 2017). The synthesis of PG starts in the cytoplasm with the formation of UDP-*N*-acetylmuramyl (UDP-MurNAc)-pentapetide and UDP-*N*-acetylglucosamine (UDP-GlcNAc) precursors (Vollmer et al., 2008a), and continues at the cell membrane with the formation of lipid I and lipid II [GlcNAc-β-(1,4)-MurNAc(pentapeptide)-pyrophosphoryl-undecaprenol] (Rogers et al., 1980; Henrich et al., 2016). Lipid II is flipped across the cytoplasmic membrane most likely by MurJ (Ruiz, 2015), FtsW/RodA (Mohammadi et al., 2014; Meeske et al., 2016), or both, although the mechanism is not yet totally defined. Penicillin binding proteins (PBPs) use lipid II to polymerize glycan chains through their glycosyltransferase (GTase) activity (Goffin and Ghuysen, 1998; Sauvage et al., 2008). These chains are then attached to the existing PG layer by transpeptidases (TPases) forming peptide cross-links (Vollmer and Born, 2010). *Escherichia coli*, transpeptidation occurs mainly by the activity of PBPs with DD-TPase activity, which carry out the formation of the peptide cross-link between D-Ala^4^ and *meso*-DAP^3^ (4–3 or DD cross-link) (Vollmer and Born, 2010). In *E. coli*, the C-terminal domain of both class A PBPs (PBP1a, PBP1b, and PBP1c) and the class B PBPs (PBP2 and PBP3) (Sauvage et al., 2008) have TPase activity. PBP1a and PBP1b are involved in both cell elongation and division (Bertsche et al., 2006), whereas the role of PBP1c is likely to be involved in a PG repair mechanism (Budd et al., 2004). PBP1a and PBP1b are the major bi-functional PBPs and at least one of these proteins is essential for the survival of the cells (Yousif et al., 1985). The enzymatic activity of PBP1a and PBP1b is stimulated by direct interaction with their cognate outer-membrane anchored lipoproteins LpoA and LpoB, respectively (Paradis-Bleau et al., 2010; Typas et al., 2010). Another regulatory protein, CpoB, specifically modulates the stimulation of the TPase of PBP1b by LpoB *in vitro*, and together with TolA couples PG synthesis with outer-membrane constriction during cell division (Gray et al., 2015).

Class B PBPs are predicted monofunctional, essential DD-TPases involved in cell elongation (PBP2) or cell division (PBP3) (Den Blaauwen et al., 2008). Class C PBPs perform DD-carboxypeptidase (DD-CPase) activity, for hydrolysis of the D-Ala^4^-D-Ala^5^ peptide bond of the peptide stems (PBP4, PBP4b, PBP5, PBP6a, and PBP6b), and/or endopeptidase (EPase) activity for the cleavage of the D-Ala^4^-*meso*-DAP^3^ bonds (PBP4, PBP7, AmpH) (Baquero et al., 1996; Broome-Smith et al., 1998; Vollmer and Holtje, 2004; Vega and Ayala, 2006; Vollmer et al., 2008b). PBP5 is the major DD-CPase in *E. coli* and its deletion results in aberrant cells and severe morphological defects that the other DD-CPases cannot compensate (Nelson and Young, 2000). Under acidic conditions, PBP6b is more active and stable *in vitro* and becomes the major DD-CPase (Peters et al., 2016).

In *E. coli*, the majority of the crosslinked stem peptides of the PG net are 4–3 cross-links, made by PBPs, but between 3 and 10% connect two *meso*-DAP^3^ residues (Glauner et al., 1988). This 3–3 cross-linking reaction is performed by LD-TPases. *E. coli* has six LD-TPases: LdtA to LdtF that were previously ErfK (A), YbiS (B), YcfS (C), YcbB (D), YnhG (E), and YafK (F) (Magnet et al., 2007, 2008; Morè et al., unpublished). The first three enzymes (LdtA–C) transfer the *meso*-DAP^3^ residue of PG stem peptides to the C-terminal Lys of Braun’s lipoprotein (Magnet et al., 2007), increasing the stability of the cell envelope, while LdtD-LdtF install 3–3 cross-links (Magnet et al., 2008; Morè et al., unpublished). Whether the redundant LD-TPases perform differently under divergent pH or other biochemical conditions, as it was observed for DD-CPases (Peters et al., 2016), remains still unknown.

β-Lactams antibiotics inhibit the DD-TPase activity of PBPs (Curtis et al., 1979; Kocaoglu and Carlson, 2015), although the subgroup of the carbapenems can inactivate both DD- and LD-TPases (Mainardi et al., 2007). In *Enterococcus faecium*, LD-TPases are able to bypass DD-TPases, leading to high level of resistance to β-lactam antibiotics (Mainardi et al., 2005). This resistance mechanism was recently reproduced in *E. coli*, where the expression of *ldtD* resulted in resistance to ampicillin after fully bypassing the DD-TPase pathway (Hugonnet et al., 2016). This resistance relied on the overproduction of LdtD, although a functional GTase domain of PBP1b and the DD-CPase activity of PBP5 were identified as required for growth in the presence of ampicillin (Hugonnet et al., 2016).

When the TPase activity of PBPs is blocked by β-lactams, GTases will continue to synthesize glycan chains that are not properly cross-linked (Park, 1995; Bertsche et al., 2005; Born et al., 2006; Banzhaf et al., 2012; Cho et al., 2014), and the still active LD-TPases may be able to bypass the DD-TPases (Hugonnet et al., 2016). Since ampicillin does not discriminate between PBPs, we investigated whether LdtD would be able to compensate for the specific activity of the essential cell division TPase PBP3. Interestingly, inhibition of PBP3 by aztreonam with simultaneous expression of *ldtD* resulted in a specific phenotype with bulges at the division site, which are absent in aztreonam-treated cells not overproducing LdtD, and reduced the level of cells lysis in treated cells with an inactive PBP1b TPase domain. This indicates that LdtD is able to compensate at least partly for the decrease in 4–3 cross-links when both PBP1b TPase domain and the essential PBP3 are blocked. To study the function of LdtD, we used the fluorescent D-amino acid (FDAA) NADA (Kuru et al., 2012) that can be incorporated in the bacterial PG likely by the activity of LD-TPases (Kuru et al., 2017). Through this method, we confirmed the role of LdtD and its partners in the incorporation of NADA as well as the function of LpoB and CpoB in regulating PBP1b activity *in vivo*. These findings would validate LdtD as new antibiotic target and could encourage the development of an assay that allows the identification of new antibiotics.

## Materials and Methods

### Strains and Reagents

Unless specified, all different compounds used for media composition were purchased from Sigma-Aldrich. NBD-amino-D-alanine (NADA) and HCC-amino-D-alanine (HADA) were synthesized according to Kuru et al. (2012). A detailed description of the strains is shown in **Supplementary Table S1**. Wild-type (WT) *E. coli* BW25113 strain was described in (Datsenko and Wanner, 2000). *E. coli* BW25113Δ(*ldtA*, *ldtB*, *ldtC*, *ldtD*, *ldtE*, *ldtF*) (BW25113Δ6LDT) was described in Kuru et al. (2017). BW25113Δ(*ldtA*, *ldtB*, *ldtC*, *ldtD*, *ldtE*, *ldtF*, *dacA*) (BW25113Δ6LDTΔdacA) was constructed by P1 phage transduction of *E. coli* BW25513Δ6LDT as described in Thomason et al. (2014). Donor lysate was prepared from strain ECK0625 (with the deletion of *dacA*) from the Keio collection (Baba et al., 2006). Single colonies were picked and checked by PCR for successful replacement of the *dacA* gene by the kanamycin resistance cassette. Positive transductants were transformed with pCP20 to remove the kanamycin cassette as described in Cherepanov and Wackernagel (1995). BW25113Δ*lpoA*, BW25113Δ*lpoB*, BW25113Δ*mrcA*, BW25113Δ*mrcB*, and BW25113Δ*cpoB* were described in Gray et al. (2015). BW25113Δ*pbpC* is from the Keio collection (Baba et al., 2006). WT CS109 and CS109Δ*dacA* are described in Denome et al. (1999). CS109Δ*dacC* and CS109Δ*dacD* are described in Potluri et al. (2012).

### Plasmid Construction

A detailed description of the plasmids is shown in **Supplementary Table S3**. pJEH12(LdtD) (Hugonnet et al., 2016) was used to construct plasmids expressing the other LD-TPase genes. pGS121, pGS124, pAMS01(LdtE), and pAMS02(LdtF) were designed as described (Morè et al., unpublished). pAMS03(LdtA), pAMS04(LdtB), and pAMS05(LdtC) were constructed using the Gibson assembly method (Gibson et al., 2009) by cloning *ldtA*, *ldtB*, and *ldtC* into pJEH12(LdtD), respectively. *ldtA*, *ldtB*, and *ldtC* genes were amplified from *E. coli* LMC500 (Taschner et al., 1988) chromosomal DNA using oligonucleotides AMS-GA7k-F/AMS-GA7k-R, AMS-GA7y-F/AMS-GA7y-R, and AMS-GA7c-F/AMS-GA7c-R, respectively (**Supplementary Table S2**). These oligonucleotides contain 24-nt overlapping arms for the pJEH12(LdtD) plasmid, upstream and downstream the *ldtD* gene. The plasmid pJEH12(LdtD) was fully linearized, except for the *ldtD* cassette, by PCR amplification using oligonucleotides AMS-GA7-F and AMS-GA7-R that anneal upstream and downstream the *ldtD* cassette. Amplified fragments were mixed and assembled by incubating them for 1 h at 50°C in Gibson assembly mix (Gibson et al., 2009).

The plasmid pSAV057 (Alexeeva et al., 2010) was used as control plasmid since it lacks a cassette for the expression of proteins involved in PG synthesis. The plasmids pWA001 (Banzhaf et al., 2012), pUM1Bα (Meisel et al., 2003), and pNM039 were used to express mCherry-PBP1a, PBP1b gene, and mCherry-PBP1c, respectively. pNM039 was constructed by cloning *pbpC* into pNM004 (Meiresonne et al., 2017). *pbpC* was amplified from chromosomal DNA with primers nm182 and nm183 containing restriction sites for *Nco*I and *Eco*RI, also used to digest pNM004. Plasmid and insert were ligated by using a T4 DNA ligase (NEB, Ipswich, MA, United States). PBP1b mutant variants were produced from pUM1BTG^∗^α (PBP1bE233Q, inactive GTase; PBP1b GT^∗^), pUM1Bα^∗^ (PBP1bS510A, inactive TPase; PBP1b TP^∗^), and pUM1BTG^∗^α^∗^ (PBP1bE233Q, S510A, with inactive GTase and TPase; PBP1b GT^∗^TP^∗^) (Meisel et al., 2003).

pNM009, pAM6a, and pAM6b (Meiresonne et al., 2017) were used for the expression of the genes of PBP5, PBP6a, and PBP6b, respectively.

### Peptidoglycan Labeling

**FIGURE 1:**
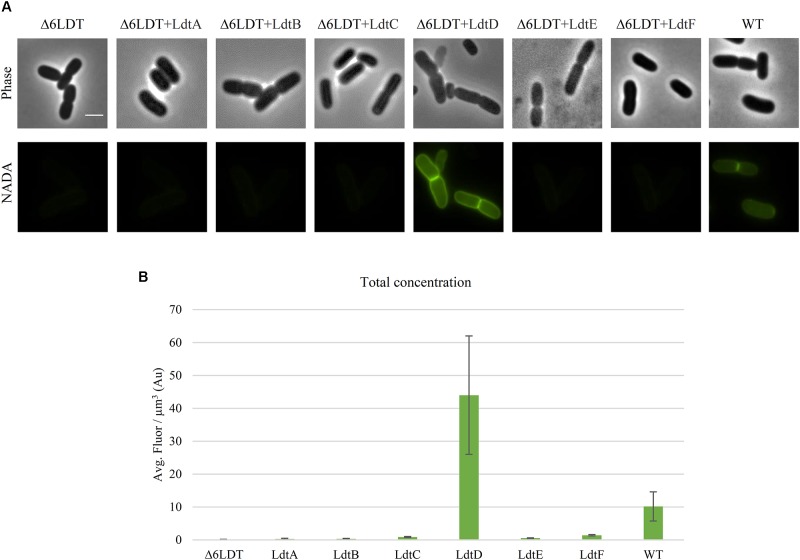
LdtD is the main LD-TPase incorporating NADA at neutral pH during exponential growth. **(A)** Phase contrast and corresponding fluorescence images of cells of BW25113Δ6LDT (control), BW25113Δ6LDT expressing *ldtA–F* (from left to right), and BW25113 in the presence of 0.5 mM NADA. Cells were grown to exponential phase in LB at 37°C. *ldtD* expression was induced for two mass doubling times with 50 μM IPTG, and the NADA pulse was for 20 min. Scale bar, 2 μm. **(B)** Quantification of the cellular concentration of incorporated NADA in the BW25113Δ6LDT strain not expressing (control) (*n* = 345) or expressing *ldtA* (*n* = 390), *ldtB* (*n* = 309), *ldtC* (*n* = 620), *ldtD* (*n* = 368), *ldtE* (*n* = 846), or *ldtF* (*n* = 423) and in BW25113 cells (*n* = 305). The values are mean ± SD of *n* number of cells.

The incorporation of FDAAs enables the analysis of real-time PG biosynthesis in growing cells, without causing any significant effect on cell growth rate (Kuru et al., 2012). LD-TPases require tetrapeptides as donor peptides, where the Ala residue at position 4 will be replaced by the FDAA. Here we chose the FDAA NADA to study its incorporation presumably by LdtD *in vivo*. **Supplementary Table S4** indicates the different strains used for PG labeling experiments. For all the experiments, cells were grown in rich medium [Lysogeny Broth (LB) or Antibiotic Broth (AB) (Sigma-Aldrich)] overnight at 37°C. The day after, samples were diluted 1:500 in pre-warmed growth medium and grown at 37°C until OD_600_ was equal to 0.25. Cells were then diluted (1:10) and the expression of *ldtD* was induced with 50 μM IPTG for two mass doubling times when the OD_600_ was 0.05. Cells were collected by centrifugation and resuspended in 100 μL pre-warmed LB or AB medium. NADA (0.5 mM) (Kuru et al., 2012) was added to the culture for 2 min at 37°C except for experiments shown in **Figure 1** in which NADA labeling was for 20 min. Cells were fixed in 70% ethanol for 10 min to prevent potential cell stress resulted from the washing steps. Cells were collected by centrifugation (5,000 rpm, 5 min) and washed three times with PBS pH 7.4 (137 mM NaCl, 2.7 mM KCl, 10 mM Na_2_HPO_4_, 1.8 mM KH_2_PO_4_) to remove the excess dye. Cells were immobilized on 1% agarose (Koppelman et al., 2004) and imaged with a Nikon Eclipse T1 microscope (Nikon Plan Fluor × 100/1.30 Oil Ph3 DLL objective) coupled to a CMOS camera (Hamamatsu Flash 4.0). Quantification of the total signal of NADA per μm^3^ average cell volume (indicated as total concentration) was performed using ImageJ with the plugin ObjectJ software (Visher et al., 2015).

When antibiotics were added to the growth medium (1 μg/mL aztreonam; 1 μg/mL cefsulodin), these were added after one mass doubling time after the addition of IPTG to allow protein production. BW25113 cells expressing or not *ldtD* were treated with 1 μg/mL cefsulodin for 60 min (approximately two mass doublings). BW25113, BW25113Δ*cpoB*, or BW25113Δ*lpoB* cells expressing or not *ldtD* were treated with 1 μg/mL aztreonam for 20 and 60 min, respectively. BW25113Δ*mrcB* cells producing PBP1b, PBP1b TP^∗^, PBP1b, GT^∗^, or PBP1b GT^∗^TP^∗^, alone or in combination with LdtD, were treated with 1 μg/mL aztreonam for 60 min. Then cells were labeled with 0.5 mM NADA for 2 min following the labeling protocol described above.

### Preparation of Lysate From Cells Overexpressing LD-TPase Genes at pH 5.0

BW25113Δ6LDT was transformed with pJEH12(LdtD), pAMS01(LdtE), pAMS02(LdtF), a control plasmid (pSAV057; Alexeeva et al., 2010), or the combination of pJEH12(LdtD) and pGS124(LdtF) or pAMS02(LdtF) and pGS121(LdtE). A single transformant was used to inoculate 5 mL of AB (Sigma-Aldrich) overnight at 37°C at pH 5.0. A 1:1000 dilution was performed in fresh AB cultures (400 mL each, in duplicate) from the overnight cultures. Samples were grown at 37°C and expression of LD-TPase genes was induced with 50 μM IPTG when the OD_600_ was 0.2. After reaching the late exponential phase (OD_600_ 0.8), samples were cooled in ice and harvested by centrifugation at 4°C. The cell pellet was resuspended in 6 mL ice-cold water and dropped slowly into 6 mL boiling 8% SDS water solution. Samples were boiled for 1 h.

### Analysis of Muropeptide Composition by HPLC

Peptidoglycan was prepared from 300 mL of cell lysate and the muropeptide composition was determined as described (Glauner et al., 1988; Bui et al., 2009). The PG was digested with cellosyl (gift from Hoechst, Frankfurt, Germany) and the resulting muropeptides were reduced with few crystals of sodium borohydride in 250 mM sodium borate buffer, pH 9.0. The reduced muropeptides were separated by HPLC and the muropeptide pattern was analyzed as described (Glauner et al., 1988).

### Protein Purification

For the purification of LdtD, *E. coli* LOBSTR-BL21(DE3) (Kerafast) cells were transformed with pETMM82, a plasmid encoding for LdtD carrying an N-terminal DsbC-His6-tag followed by a TEV-protease cleavage site (Hugonnet et al., 2016), and grown at 30°C in 1 L of TB medium (Tartof and Hobbs, 1987) (supplemented with 5 mM MgCl_2_ and 5 mM MgSO_4_) until OD_600_ 0.3. LdtD overproduction was induced by adding IPTG (Generon) to a final concentration of 0.5 mM. Cells were incubated for 19 h at 16°C and harvested by centrifugation for 15 min at 4,500 rpm and 14°C. The resulting cell pellet was resuspended in 60 mL buffer A (20 mM Tris pH 8.0, 1 M NaCl, 10 mM imidazole) supplemented with 1 mM phenylmethyl sulfonyl fluoride (Sigma-Aldrich), 1× protease inhibitor cocktail (Sigma-Aldrich), and deoxyribonuclease I (Sigma-Aldrich). Cells were broken by sonication and centrifuged for 1 h at 130,000 × *g* at 4°C. The supernatant was recovered, mixed with 0.5 mL Ni-NTA Superflow (Qiagen) preequilibrated in buffer A (supplemented with 10 mM imidazole), and incubated under continuous gentle stirring at 4°C. After 1.5 h another 0.5 mL of Ni-NTA Superflow (Qiagen) was added and incubated for 1.5 h. The suspension was poured in a gravity flow column and washed two times with 20 column volumes (CV) buffer B (20 mM Tris/HCl pH 7.0, 150 mM NaCl) supplemented with 20 mM imidazole, 5 mM ATP, and 1 mM MgCl_2_ to remove tightly bound chaperone proteins. After three more washing steps with 20 CV of buffer B each (2× 40 mM imidazole, 1× 50 mM imidazole), the protein was eluted with buffer B supplemented with 300 mM imidazole and glycerol was added to the elution fractions to a final concentration of 10%. The protein was dialyzed against 2 × 2 L dialysis buffer 1 (25 mM Tris pH 7.0, 300 mM NaCl, 10% glycerol) for 1 h each at 4°C. The protein solution was supplemented with 5 mM β-mercaptoethanol (Sigma-Aldrich), 10 U/mL TEV-protease (Promega), and dialyzed against 1 L of dialysis buffer 2 (25 mM Tris pH 7.0, 300 mM NaCl, 5 mM β-mercaptoethanol, and 10% glycerol) for 1 h and against an additional 1 L overnight at 4°C.

The sample was mixed with 1 mL of Ni-NTA-agarose preequilibrated in dialysis buffer 2 containing 50 mM of imidazole and incubated for 2–3 h at 4°C under gentle stirring. The suspension was poured in a gravity flow column and the DsbC-His-tag-free protein present in the flow through was further purified by size exclusion chromatography on a HiLoad 26/60 Supedex 200 (GE Healthcare) column using size exclusion buffer (25 mM Tris/HCl pH 7.5, 300 mM NaCl, 10% glycerol) and a flow rate of 1 mL/min. Purity was determined by SDS–PAGE and combined fractions were concentrated and stored in aliquots at -80°C.

PBP1b-TP^∗^ was purified as described in Typas et al. (2010); LpoB was purified as described in Egan et al. (2014); PBP5 was purified as described in Peters et al. (2016).

### HADA Incorporation Assay With Muropeptides

Assays were carried out in a final volume of 50 μL containing 25 mM Tris/HCl pH 7.0, 100 mM NaCl, 10 mM MgCl_2_, 0.1% Triton X-100, 200 μM HADA, 2 μM LdtD, and muropeptides (∼100 μg) obtained by digesting PG from BW25113Δ6LDT with cellosyl. The sample was incubated at 37°C overnight and the reaction was stopped by boiling for 10 min. Muropeptides were reduced and analyzed by HPLC as described (Glauner et al., 1988).

### HADA Incorporation Coupled to PG Synthesis

Assays were carried out in a final volume of 50 μL containing 50 mM Tris–HCl pH 7.0, 175 mM NaCl, 10 mM MgCl_2_, 0.1% Triton X-100, 200 μM HADA, radioactively labeled lipid II (10,000 dpm) (Bertsche et al., 2005), 15 μL of PG from BW25113Δ6LDT, 1 μM PBP5, 1 μM PBP1b-TP^∗^, 2 μM LpoB, and 2 μM LdtD. The reaction mixture was incubated for 2 h at 37°C. The reaction was stopped by boiling the samples for 10 min.

Samples were centrifuged for 20 min, the supernatant recovered, and adjusted to pH 4 with 20% phosphoric acid. HPLC analysis was carried out as described (Bertsche et al., 2005). Muropeptides were detected by online radioactivity detector and absorbance at 205 nm.

## Results

### LdtD Is Required to Incorporate NADA at Neutral pH

DD- and LD-TPases have been proposed as the enzymes responsible for incorporation of FDAAs at the fourth position of the PG peptide stem in Gram-negative species (Kuru et al., 2015, 2017). We used the green FDAA NADA (Kuru et al., 2012) to label the PG of WT BW25113 and a strain lacking all LD-TPases BW25113Δ6LDT (Kuru et al., 2017). After a 20 min labeling pulse, the strain BW25113 was labeled but BW25113Δ6LDT did not show any PG labeling (**Figures 1A,B**) despite the presence of DD-TPases in both strains, indicating that at least one of the LD-TPases is involved in the incorporation of NADA under these conditions.

To determine which LD-TPase is responsible for NADA incorporation, BW25113Δ6LDT was transformed with plasmids expressing the different LD-TPase genes. After a 20-min pulse with NADA, only cells expressing *ldtD* incorporated NADA (**Figure 1A**). The total concentration of incorporated NADA was calculated and summarized in **Figure 1B**.

### *In vitro* Activity of LdtD for FDAA Incorporation

We also observed that purified LdtD was able to incorporate HADA, another FDAA (Kuru et al., 2012), into muropeptides (**Supplementary Figure S1**). The decrease in TetraTri(3–3) in the presence of HADA (comparing the top two chromatograms in **Supplementary Figure S1A**) might be due to competition between LD-TPase and FDAA exchange reactions. Incubating LdtD with radioactive labeled lipid II, PG from BW25113Δ6LDT, PBP5, a TPase inactive PBP1bTP^∗^, and LpoB lead to the incorporation of HADA *in vitro* (**Supplementary Figure S1B**), confirming the cooperation observed *in vivo* between LdtD and other cell division proteins.

### Effect of pH on 3–3 Cross-Linking Activity

**FIGURE 2:**
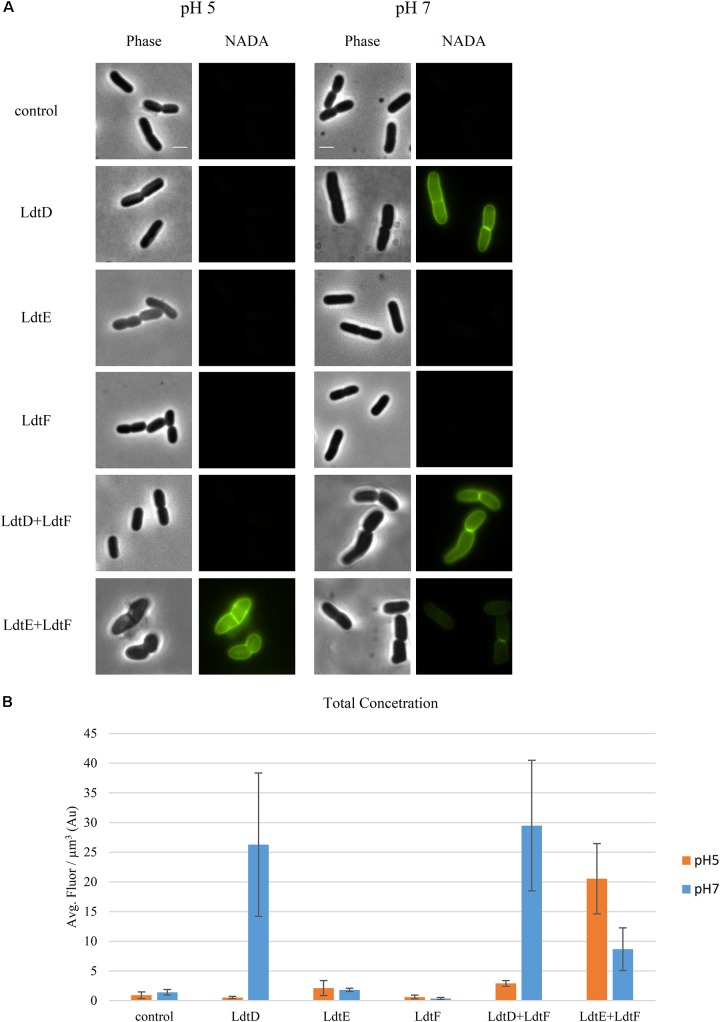
Incorporation of NADA is pH dependent. **(A)** Phase contrast and corresponding fluorescence images of BW25113Δ6LDT cells not expressing (control) or expressing *ldtD*, *ldtE*, *ldtF*, *ldtD* and *ldtF*, or *ldtE* and *ldtF* at pH 5.0 or 7.0, and labeled with NADA. Cells were grown to exponential phase in LB at 37°C. LD-TPase genes expression was induced for two mass doubling with 50 μM IPTG, and the NADA pulse was for 2 min. Scale bar, 2 μm. **(B)** Concentration of NADA in cells of BW25113Δ6LDT strain not expressing (control) (*n* = 1000 at pH 5; *n* = 1216 at pH 7) or expressing *ldtD* (*n* = 903 at pH 5.0; *n* = 1329 at pH 7.0), *ldtE* (*n* = 768 at pH 5.0; *n* = 2116 at pH 7.0), *ldtF* (*n* = 331 at pH 5.0; *n* = 1335 at pH 7.0), *ldtD* and *ldtF* (*n* = 294 at pH 5; *n* = 518 at pH 7), or *ldtE* and *ldtF* (*n* = 444 at pH 5.0; *n* = 530 at pH 7.0). The values are mean ± SD of *n* number of cells.

We evaluated the incorporation of NADA in the PG of BW25113Δ6LDT cells expressing *ldtD*, *ldtE*, *ldtF*, *ldtD*+*ldtF*, or *ldtE*+*ldtF* at both pH 5.0 and 7.0 to verify whether their LD-TPase activity has a pH preference. BW25113Δ6LDT did not show NADA incorporation in the PG of cells grown at either pH (**Figures 2A,B**). At pH 7.0, the signal of NADA incorporation was restored only when *ldtD* was mildly overproduced, in either the presence or absence of *ldtF* (**Figure 2A**). Co-expressing *ldtD* and *ldtF* lead to a higher fluorescent signal (**Figure 2B**) compared to the expression of *ldtD* alone. Expressing *ldtE* alone or in combination with *ldtF* did not show NADA incorporation in the PG of BW25113Δ6LDT grown at pH 7.0 (**Figure 2A**).

At pH 5.0, NADA was incorporated in the PG of the BW25113Δ6LDT when LdtE and LdtF were overproduced together (**Figure 2A**). Expressing *ldtD* alone or in combination with *ldtF* did not show PG labeling at pH 5.0 (**Figure 2A**).

**FIGURE 3:**
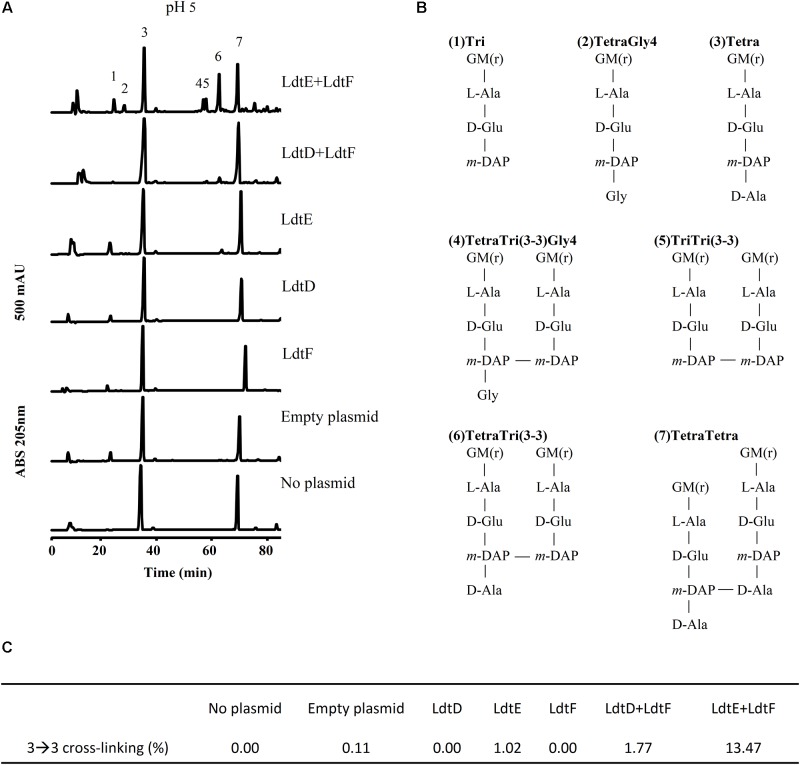
LD-TPase-mediated formation of 3–3 cross-links in cells growing at acidic pH. **(A)** Muropeptide profiles of BW25113Δ6LDT cells containing either no plasmid, control plasmid pSAV057, or plasmid for expression of *ldtD*, *ldtE*, *ldtF*, *ldtE* and *ldtF*, or *ldtD* and *ldtF*, grown in the presence of 50 μM IPTG at pH 5.0. **(B)** Structures of major muropeptides numbered in the top chromatogram in panel **A**. LD-TPase products are muropeptides containing 3–3 cross-links (No. 4–6), tripeptides (peaks 1 and 5), and glycine at position 4 (Gly4, No. 2 and 4). G, *N*-acetylglucosamine; M(r), *N*-acetylmuramitol; L-Ala, L-alanine; D-Glu, D-glutamic acid; D-Ala, D-alanine; *meso*-DAP, meso-diaminopimelic acid. **(C)** Relative amount (%) of muropeptides with 3–3 cross-links.

To confirm the pH preference of LdtE and LdtF observed *in vivo*, we analyzed the muropeptide composition of the PG of BW25113Δ6LDT expressing *ldtD*, *ldtE*, and/or *ldtF* at pH 5.0. As expected, BW25113Δ6LDT did not contain muropeptides with 3–3 cross-links (**Figures 3A,C**). The PG of BW25113Δ6LDT expressing *ldtD* did not show any 3–3 cross-links (**Figures 3A,C**) but the co-expression of *ldtD* and *ldtF* in cells growing at pH 5.0 showed a small portion of muropeptides with 3–3 cross-links (**Figures 3A,C**).

LdtE appeared to be weakly active in cells growing at pH 5.0, where 1.02% of the muropeptides contained 3–3 cross-links (**Figures 3C**). However, co-expression of *ldtF* with *ldtE* in cells growing at pH 5.0 increased the content of muropeptides with 3–3 cross-links from 1.02 to 13.47% (**Figure 3C**). LdtE and LdtF may cooperate with each other synergistically.

### NADA Incorporation by LdtD and Class A PBPs

**FIGURE 4:**
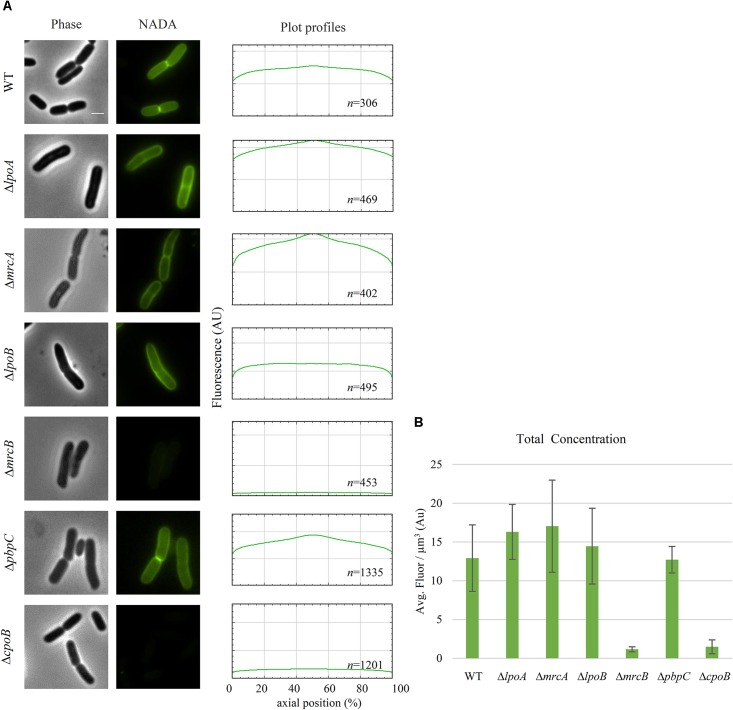
PBP1b is required for NADA incorporation by LdtD. **(A)** Phase contrast and corresponding fluorescence images of cells of BW25113, BW25113Δ*lpoA*, BW25113Δ*mrcA* (encoding PBP1a), BW25113Δ*lpoB*, BW25113Δ*mrcB* (PBP1b), BW25113Δ*pbpC* (PBP1c), and BW25113Δ*cpoB* expressing *ldtD* and labeled with 0.5 mM NADA. Scale bar, 2 μm. The right panel shows the fluorescence profiles per average cell (from 0 to 1000 AU) plotted against normalized cell length (from 0 to 100%). *n* represents the number of cells analyzed. **(B)** Concentration of NADA fluorescence (fluorescence signal per μm^3^ average cell volume). Quantification of the incorporated NADA in the WT strain, BW25113Δ*lpoA*, BW25113Δ*mrcA*, BW25113Δ*lpoB*, BW25113Δ*mrcB*, BW25113Δ*pbpC*, and BW25113Δ*cpoB* strains expressing *ldtD*. The values are mean ± SD of *n* number of cells. *n* is indicated on the fluorescence profiles.

The NADA incorporation activity of LdtD was evaluated after deletion of a gene encoding a Class A PBP (PBP1a, PBP1b, or PBP1c) or their regulators (LpoA, LpoB, or CpoB). NADA incorporation was detected in the lateral wall and at mid-cell in BW25113Δ*mrcA* and in BW25113Δ*lpoA* strains expressing *ldtD* with similar intensity, whereas a slightly lower NADA concentration was observed for BW25113Δ*pbpC* cells expressing *ldtD* (**Figures 4A,B**). BW25113Δ*mrcB* expressing *ldtD* did not incorporate NADA indicating that PBP1b is needed for NADA incorporation by LdtD. Surprisingly, *ldtD* expression in BW25113Δ*lpoB* cells showed a clear labeling of the lateral wall but the signal at mid-cell was lost or weak in comparison with BW25113Δ*lpoA* cells (**Figures 4A,B**). This suggests that the mid-cell incorporation of NADA is due to the local activation of PBP1b by LpoB. To confirm this, BW25113 cells expressing *ldtD* were treated with 1 μg/mL cefsulodin, since this antibiotic is specific for PBP1a and PBP1b (Curtis et al., 1979; Sarkar et al., 2012; Kocaoglu and Carlson, 2015). NADA was incorporated in the lateral wall but clear empty septa (non-labeled) were observed (**Supplementary Figure S3**), confirming that the inhibition of PBP1b affected the NADA-incorporation activity of LdtD at mid-cell, as in the case of the BW25113Δ*lpoB* strain. The deletion of *cpoB* did not allow the incorporation of NADA by LdtD (**Figure 4A**).

**FIGURE 5:**
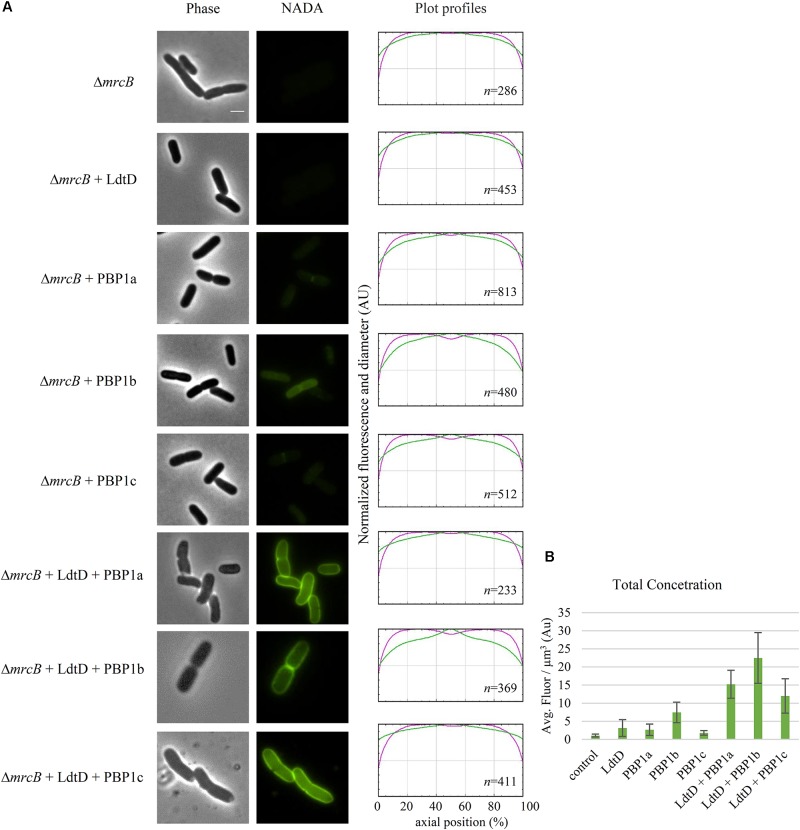
Effect of plasmid-expressed genes encoding class A PBPs on NADA incorporation by LdtD. **(A)** Phase contrast and corresponding fluorescence images of BW25113Δ*mrcB* (without PBP1b) (control) expressing or not *ldtD*, *mrcA*, *mrcB*, *pbpC*, *ldtD* and *mrcA*, *ldtD* and *mrcB*, or *ldtD* and *pbpC*, labeled with a 0.5 mM NADA pulse of 2 min. Scale bar, 2 μm. The right panel shows the average normalized fluorescence profiles (green) and diameter (purple) per cell (from 0.0 to 1.0) plotted against normalized cell length (from 0 to 100%). *n* represents the number of cells analyzed. **(B)** Concentration is the NADA fluorescence (signal per μm^3^ average cell volume). Quantification of the incorporated NADA in cells of BW25113Δ*mrcB* (control) or expressing *ldtD*, *mrcA*, *mrcB*, *pbpC*, *ldtD* and *mrcA*, *ldtD* and *mrcB*, and *ldtD* and *pbpC*. The values are mean ± SD of *n* number of cells. *n* is indicated on the fluorescence profiles.

Because PBP1b was found to be needed for NADA incorporation by LdtD, BW25113Δ*mrcB* was co-transformed with pJEH12(LdtD) and a plasmid expressing PBP1a, PBP1b, or PBP1c genes to determine whether any of these GTases could compensate for the absence of PBP1b. Surprisingly, co-expression of LdtD and PBP1a or PBP1c restored the signal of PG labeling, with no differences in total NADA concentration, but with the lack of NADA incorporation at mid-cell (**Figures 5A,B**). As expected, the co-expression of the genes encoding PBP1b and LdtD restored the signal not only at the lateral wall but also at mid-cell (**Figure 5A**), confirming that PBP1b is required for NADA incorporation by LdtD, especially at mid-cell.

### NADA Incorporation Requires Class C PBPs

**FIGURE 6:**
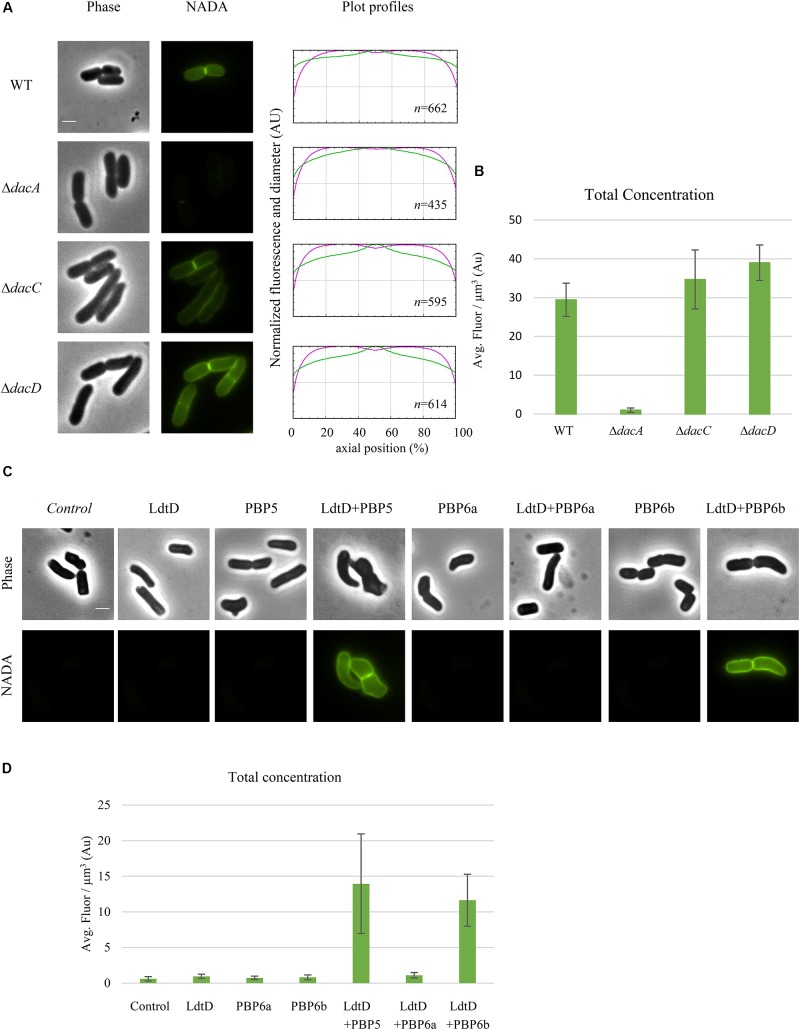
Class C PBP activity is required for NADA incorporation by LdtD. **(A)** Phase contrast and fluorescence images of CS109, CS109Δ*dacA* (PBP5) expressing *ldtD*, CS109Δ*dacC* (PBP6a) expressing *ldtD*, and CS109Δ*dacD* (PBP6b) expressing *ldtD* labeled with a 0.5 mM NADA pulse of 2 min. Scale bar, 2 μm. The right panel shows the average fluorescence profiles (green) and diameter (purple) per cell (from 0.0 to 1.0) plotted against normalized cell length (from 0 to 100%). *n* represents the number of cells analyzed. **(B)** Total concentration is the NADA fluorescence (signal per μm^3^ average cell volume). Quantification of the incorporated NADA in the CS109 strain, CS109Δ*dacA* expressing *ldtD*, CS109Δ*dacC* expressing *ldtD*, and CS109Δ*dacD* expressing *ldtD*. The values are mean ± SD of *n* number of cells. **(C)** Phase contrast and fluorescence images of BW5113D6LDTD*dacA* cells not expressing (control) or expressing *ldtD*, *dacA*, *ldtD* and *dacA*, *dacC*, *ldtD*, and *dacC*, *dacD*, or *ldtD* and *dacD*, labeled with 0.5 mM NADA. Scale bar, 2 μm. The right panel shows the average fluorescence profiles (green) and diameter (purple) per cell (from 0.0 to 1.0) plotted against normalized cell length (from 0 to 100%). **(D)** Quantification of the total concentration of incorporated NADA in the BW25113Δ6LDTΔ*dacA* cells not expressing (control) or expressing *ldtD*, *dac*A, *ldtD* and *dacA*, *dacC*, *ldtD*, and *dacC*, *dacD*, or *ldtD* and *dacD*. The values are mean ± SD of *n* number of cells.

To determine whether a DD-CPase is required for the NADA incorporation activity of LdtD, CS109Δ*dacA* (lacking PBP5), CS109Δ*dacC* (PBP6a), and CS109Δ*dacD* (PBP6b) overproducing LdtD were labeled with NADA. Deletion of *dacA* did not enable LdtD to incorporate NADA in the PG (**Figure 6A**), whereas the deletion of *dacC* and *dacD* allowed the labeling of cells with NADA (**Figures 6A,B**). This indicates that PBP5 is required for the incorporation of NADA by LdtD.

BW25113Δ6LDTΔdacA was co-transformed with pJEH12(LdtD) and a plasmid expressing the genes of PBP5, PBP6a, or PBP6b to determine whether any of these DD-CPases could complement for the absence of PBP5. As expected, the combined expression of *ldtD* and *dacA* restored the signal of NADA incorporation (**Figure 6C**). Interestingly, co-expression of *ldtD* and the gene encoding PBP6b also complemented for the absence of PBP5 (**Figure 6C**) yielding similar NADA incorporation at mid-cell compared to the co-expression of *dacA* and *ldtD* (**Figures 6C,D**). Co-expressing *ldtD* and the gene encoding PBP6a did not allow for LdtD-mediated incorporation of NADA (**Figure 6C**) meaning that PBP6a, unlike PBP6b, is not able to compensate for the absence of PBP5.

### Effect of Aztreonam on Cells Expressing *ldtD*

**FIGURE 7:**
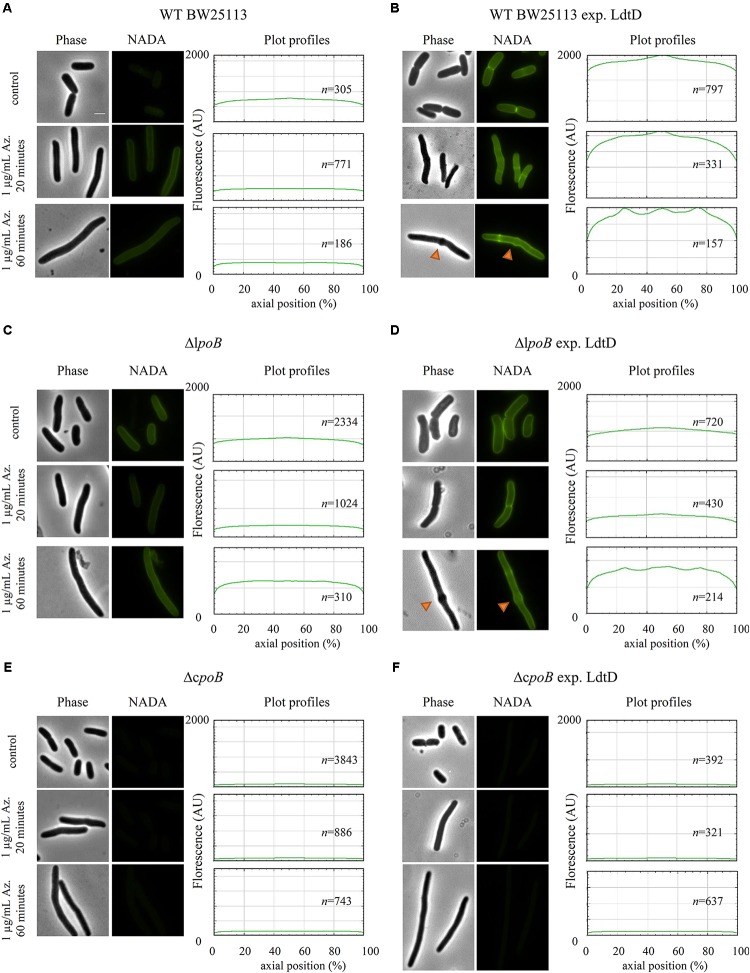
*ldtD* expression in aztreonam-treated cells creates bulges at mid-cell. Phase contrast images, corresponding fluorescent images, and fluorescence profiles per average cell (from 0 to 2000 AU) plotted against normalized cell length (from 0 to 100%). **(A)** BW25113, **(B)** BW25113 expressing *ldtD*, **(C)** BW25113Δ*lpoB*, **(D)** BW25113Δ*lpoB* expressing *ldtD*, **(E)** BW25113Δ*cpoB*, and **(F)** BW25113Δ*cpoB* expressing *ldtD*. Upper panel, no antibiotic; middle panel, 20 min incubation with 1 μg/mL aztreonam; lower panel, 60 min of incubation with 1 μg/mL aztreonam. PG was labeled with 0.5 mM NADA with a 2 min labeling pulse after the 20 or 60 min incubation with aztreonam. Scale bar, 2 μm. Red triangles point to mid-cell bulges. *n* represents the number of cells analyzed.

We wanted to determine whether the overproduced LdtD could compensate for the block of the essential cell division class B PBP3. We treated BW25113 cells expressing or not *ldtD* with aztreonam for 20 and 60 min and then labeled them with NADA. We observed NADA incorporation at the lateral wall and mid-cell after the 20 min pulse (**Figures 7A,B** mid-panel). After 60 min, cells overexpressing *ldtD* showed bulges at the previous division site (**Figure 7B** low-panel) while cells not expressing *ldtD* grew as smooth filaments (**Figure 7A** low-panel), indicating that the expression of *ldtD* is responsible for this bulging phenotype. Moreover, NADA incorporation was detected at one-fourth and three-fourth of the filaments (**Figure 7B** low-panel), presumably at preseptal sites. Similar results were observed for the BW25113Δ*lpoB* cells expressing *ldtD* (**Figures 7C,D** mid- and lower-panel). In contrast, the BW25113Δ*cpoB* strain grown in the presence of aztreonam showed smooth filaments and NADA was not incorporated irrespective whether *ldtD* was expressed or not (**Figures 7E,F**).

Because of the importance of PBP1b for the function of LdtD, we repeated the treatment with 1 μg/mL aztreonam for 1 h in BW25113Δ*mrcB* cells either expressing genes encoding different PBP1b variants (WT PBP1b, PBP1b GT^∗^, PBP1b TP^∗^, and PBP1b GT^∗^TP^∗^) alone or co-expressed with *ldtD*. In the absence of *ldtD* expression, cells readily lysed in all cases except for the expression of the WT PBP1b gene, which led to filaments with bulges at mid-cell (**Supplementary Figure S4**). A similar level of lysis was observed when *ldtD* was co-expressed with the genes encoding PBP1b GT^∗^ or PBP1b GT^∗^TP^∗^. Interestingly, the level of lysis was considerably lower when *ldtD* was co-expressed with the gene encoding PBP1b TP^∗^ (**Supplementary Figure S4**).

## Discussion

### pH Dependence for FDAAs Incorporation by LdtD

Penicillin-insensitive LD-TPases were proposed to be responsible for the incorporation of exogenous D-Met, other D-amino acids (Tsuruoka et al., 1984; Pisabarro et al., 1985; Caparrós et al., 1992) and FDAA probes, such as NADA or HADA (Kuru et al., 2012) into the terminal position of tetrapeptide stems in *E. coli*. Here we aimed to identify which of the six LD-TPases of *E. coli* incorporates NADA. NADA was readily incorporated into BW25113 cells but not those of a strain lacking all LD-TPase genes (BW25113Δ6LDT), indicating that at least one of the LD-TPs is required for the PG labeling by LD-TPases. In the background of the BW25113Δ6LDT strain, NADA incorporation was only visible when LdtD was overproduced from a plasmid (**Figure 1**). Because cells were grown in exponential phase, LdtD might be more active in exponentially growing cells, or has a higher NADA-incorporation activity than the other LD-TPases catalyzing 3–3 cross-links, LdtE and LdtF (Magnet et al., 2008; Morè et al., unpublished). It cannot be excluded that PBPs can incorporate NADA, which is then immediately removed by DD-CPases. However, since NADA incorporation was not observed in the BW25113Δ6LDTΔdacA, the main DD-CPase PBP5 is not needed for the removal. We also observed that LdtD was able to incorporate the FDAA HADA *in vitro* (**Supplementary Figure S1**). From both *in vivo* and *in vitro* studies, we can conclude that FDAAs are a suitable substrate for LdtD.

A recent study found that PBP6b is more active at pH 5.0 (compared to pH 7.5), whereas PBP5 and PBP6a showed a lower activity at acidic pH, both in the cell and with the purified enzymes (Peters et al., 2016). Presumably, *E. coli* maintains sets of PG enzymes to grow robustly at different growth conditions, such as different pH values (Pazos et al., 2017). LdtD was not able to incorporate NADA at pH 5.0, whereas LdtE seemed to be inactive at pH 7.0 and weakly active at pH 5.0 (**Figure 2**). Nevertheless, LdtE and LdtF were observed to be more active when they were overproduced together at acidic pH (**Figures 2**, **3**). They may be active under acidifying condition such as fermentation while LdtD would be active during respiratory growth at near neutral pH. Why co-expression of both *ldtE* and *ldtF*, and not *ldtD* and *ldtF*, improved the incorporation of NADA at pH 5.0 remains still unclear but it suggests the possibility of a potential activation system between these two LD-TPases.

### PBP1b, LpoB, and CpoB Are Required for Efficient Incorporation of NADA by LdtD

LdtD can work on old, pre-formed PG (**Supplementary Figure S1**) but may also work on newly synthesized PG in the context of an ampicillin resistant mutant strain of *E. coli* (Hugonnet et al., 2016). Indeed, we found here that the incorporation of NADA by overproduced LdtD requires the presence of PBP1b and its regulators LpoB and CpoB (**Figure 4A**). The absence of PBP1a, PBP1c, or LpoA did not affect the NADA incorporation by LdtD, since we observed PG labeling in the BW25113Δ*lpoA*, BW25113Δ*mrcA*, and BW25113Δ*pbpC* strains whether (**Figure 4A**) or not (**Supplementary Figure S2**) *ldtD* was expressed. These data align well with previous work showing that LdtD is functionally linked to PBP1b but not PBP1a (Hugonnet et al., 2016). In the BW25113Δ*mrcB* strain, PBP1a and PBP1c did not compensate for the absence of PBP1b to enable LdtD-mediated NADA incorporation, unless they were overproduced from plasmid together with LdtD (**Figure 5A**). Co-overproducing LdtD and PBP1a or PBP1c restored the signal of incorporated NADA only in the lateral wall but not at mid-cell. This finding supposes the first phenotype associated with PBP1c activity. PBP1c might have a role in situations where extra GTase activity is required, as previously hypothesized (Budd et al., 2004).

Expression of *ldtD* in BW25113Δ*lpoB* affected incorporation of NADA at mid-cell (**Figure 4A**), although the labeling at the lateral wall was comparable to the BW25113Δ*lpoA* strain (**Figures 4A,B**). LpoB showed enhanced localization at mid-cell (Typas et al., 2010) where it would activate both GTase and TPase activities of PBP1b (Egan et al., 2014). Treating BW25113 cells with cefsulodin, which specifically inhibits PBP1a and b (Curtis et al., 1979; Sarkar et al., 2012; Kocaoglu and Carlson, 2015), provided similar results to the deletion of *lpoB*. Together, these results suggest that the activation of PBP1b by LpoB is required for incorporation of NADA at mid-cell by LdtD.

CpoB modulates the stimulation of the TPase activity of PBP1b (Gray et al., 2015). Deletion of *cpoB* renders cells more sensitive to cefsulodin, indicating that the TPase activity of PBP1b strongly contributes to PG synthesis (Gray et al., 2015). Consistent with the hyper TPase activity of PBP1b, NADA was presumably not incorporated in a BW25113Δ*cpoB* strain expressing *ldtD* because of the lack of available substrate (**Figure 4A**). The results confirm and extend the role of CpoB as a regulator of the TPase activity of PBP1b *in vivo*.

### PBP6b Can Complement the Absence of PBP5 for LdtD Activity

Deletion of *dacA*, encoding the major DD-CPase PBP5, did obstruct the incorporation of NADA into PG by LdtD (**Figure 6A**). PBP5 hydrolyzes the D-Ala^4^-D-Ala^5^ bond of pentapeptide stems (Hugonnet et al., 2016), which results in the production of tetrapeptides, which are the substrate for LD-TPases (Magnet et al., 2008). Deletion of *dacC* (encoding PBP6a) and *dacD* (encoding PBP6b) allowed incorporation of NADA by overproduced LdtD and no significant differences were found between the two deletion strains (**Figure 6**). This was expected for PBP6b considering that its gene is known to be hardly expressed during exponential growth (Li et al., 2014; Peters et al., 2016), and also for PBP6A for which no *in vivo* activity has been reported (Meiresonne et al., 2017).

To evaluate the possible complementation for the absence of PBP5, LdtD and one of the class C PBPs (PBP5, PBP6a, or PBP6b) were co-overproduced in a strain lacking all LD-TPs and PBP5 (BW25113Δ6LDTΔdacA). PBP5 is the major DD-CPase in *E. coli* and the morphological defects associated with deletion of the corresponding gene cannot be compensated by the other class C PBPs present on the chromosome (Nelson and Young, 2000). However, our studies show that overproduction of PBP6b provided sufficient DD-CPase activity in a Δ*dacA* background to support LdtD-mediated incorporation of NADA.

### Expressing *ldtD* Causes Bulges in Cells Treated With Aztreonam

Recently, we proved that LdtD is able to bypass the DD-TPase pathway in cells treated with ampicillin (Hugonnet et al., 2016). We observed that NADA was strongly incorporated at mid-cell and the resistant *E. coli* was able to divide, which suggested the involvement of the essential cell division class B PBP3 TPase activity. We decided to evaluate whether overproducing LdtD could compensate for the decrease of 4–3 cross-linking derived from the inhibition of the DD-TPase activity of PBP3 by aztreonam (Sykes and Bonner, 1985). These cells have complete division machineries at potential division sites at least for two mass doublings (Pogliano et al., 1997; van der Ploeg et al., 2013). The filaments showed bulges at mid-cell after 60 min aztreonam treatment (approximately two mass doublings) in WT BW25113 cells expressing *ldtD*, indicating that the expression of *ldtD* may be responsible for this phenotype. This phenotype was also observed in ampicillin-resistant *E. coli* expressing *ldtD* (Hugonnet et al., 2016). Interestingly, bulges were also present in the BW25113Δ*lpoB* strain overproducing *ldtD* (**Figures 7C,D**), whereas with active PBP3 the BW25113Δ*lpoB* strain did not incorporate NADA at mid-cell (**Figure 4A**). Consequently, the NADA incorporation in aztreonam-inhibited BW25113Δ*lpoB* cells likely corresponds to lateral wall synthesis, which would support the notion that PBP1b is not necessarily part of the divisome but can be active at its periphery (Cho et al., 2016).

Why does the overexpression of *ldtD* in aztreonam treated cells result in bulges? After PBP3 is inhibited by β-lactams the division machinery remains assembled for at least two mass doublings (Pogliano et al., 1997; van der Ploeg et al., 2013). The cells synthesize only PG for elongation, switching between phases of pre-septal PG synthesis and dispersed, lateral PG synthesis. After blocking PBP3 with aztreonam, the incorporation of lipid-II in the PG layer is regulated by PBP1b. Perhaps the endogenous LdtD activity in *E. coli* is not enough to compensate for the decrease in 4–3 cross-links and to incorporate GTase-derived glycan chains into PG when the essential DD-TPase PBP3 is blocked by aztreonam. However, overproduction of LdtD results in extra 3–3 cross-links that may lead to an improperly orientated septal PG synthesis and subsequent bulge formation. The presence of bulges at mid-cell is common in *E. coli* cells treated with other β-lactams, from low concentrations of penicillin (Schwarz et al., 1969) to high concentrations of ampicillin (Hugonnet et al., 2016).

We also evaluated the effect of expressing *ldtD* when the essential DD-TPase PBP3 is blocked by aztreonam and an inactive PBP1b is overproduced, since we have detected a dependence of LdtD on PBP1b. The overproduction of WT PBP1b is non-lethal for the cells in contrast to the production of inactive forms of PBP1b that cause cells to lyse (**Supplementary Figure S4**; Meisel et al., 2003). Overproducing LdtD with PBP1b TP^∗^ compensates for the loss in 4–3 cross-links, preventing and/or delaying cell lysis and lead to the observed survival phenotype.

With this work, we provide additional information on the role of LdtD in β-lactam-treated cells. These observations imply that inhibition of LD-TPases is a possible option to keep β-lactam resistance at bay.

## Author Contributions

AMS and TB conceived the experiments. AMS, CO, and JB performed the experiments and prepared figures. MV supplied NADA and HADA. EB supplied lipid II for *in vitro* studies of LdtD. AMS, TB, WV, and CO wrote the manuscript. All authors reviewed the manuscript.

## Conflict of Interest Statement

The authors declare that the research was conducted in the absence of any commercial or financial relationships that could be construed as a potential conflict of interest.
