# Supplementary material for: The Fluorescent D-Amino Acid NADA as a Tool to Study the Conditional Activity of Transpeptidases in Escherichia coli
Source: Front Microbiol. 2018 Sep 4;9:2101. doi: 10.3389/fmicb.2018.02101 (PMC6131605; doi:10.3389/fmicb.2018.02101)
Supplement: Supplementary file 1 [file Data_Sheet_1.pdf]

Supplementary Information

The fluorescent D-amino acid NADA as a tool to study the conditional activity of transpeptidases in *Escherichia coli*

Alejandro Montón Silva[1], Christian Otten[2], Jacob Biboy[2], Eefjan Breukink[3], Michael VanNieuwenhze[4], Waldemar Vollmer[2], and Tanneke den Blaauwen[1*].

[1] Bacterial Cell Biology & Physiology, Swammerdam Institute for Life Sciences, University of Amsterdam, Science park 904, 1098 XH Amsterdam, The Netherlands.

[2] The Centre for Bacterial Cell Biology, Institute for Cell and Molecular Biosciences, Newcastle University, Newcastle upon Tyne, United Kingdom.

[3] Department of Membrane Biochemistry and Biophysics, Institute of Biomembranes , Utrecht University , Padualaan 8 , 3584 CH Utrecht , The Netherlands.

[4] Department of Chemistry, Indiana University Bloomington, Bloomington, IN 47405, USA.

[*] CORRESPONDENCE:

Dr. Tanneke den Blaauwen

t.denblaauwen@uva.nl

Supplemental Information

Table S1: *E. coli* strains

Table S2: Oligonucleotides

Table S3: Plasmids

Table S4: Transformations for PG labelling with NADA

Figure S1: *in vitro* assay of HADA incorporation by LdtD at pH 7.0

Figure S2: NADA incorporation in cells expressing or not LdtD.

Figure S3: Wild type BW25113 expressing LdtD in the presence of cefsulodin.

Figure S4: BW25113Δ*mrcB* strain expressing PBP1b variants alone or with LdtD in the presence of aztreonam.

References

Table S1: *E. coli* strains

Strain	Description	Reference
BW25113	*F-, DE(araD-araB)567, lacZ4787(del)::rrnB-3, LAM-, rph-1, DE(rhaD-rhaB)568, hsdR514*	Datsenko et al., 2000
BW25113Δ*lpoA*	BW25113 Δ*lpoA*::*kan*	Gray et al., 2015
BW25113Δ*mrcA*	BW25113 Δ*mrcA*::*kan*	Gray et al., 2015
BW25113Δ*lpoB*	BW25113 Δ*lpoB*::*kan*	Gray et al., 2015
BW25113Δ*mrcB*	BW25113 Δ*mrcB*::*kan*	Gray et al., 2015
BW25113Δ*cpoB*	BW25113 Δ*cpoB*::*kan*	Gray et al., 2015
BW25113Δ*pbpC*	BW25113 Δ*pbpC*::*kan*	Baba et al., 2006
CS109	W1485 rpoS rph	Denome et al., 1999
CS109Δ*dacA*	CS109 Δ*dacA*::*kan*	Denome et al., 1999
CS109Δ*dacC*	CS109 Δ*dacC*::*kan*	Potluri et al., 2012
CS109Δ*dacD*	CS109 Δ*dacD*::*kan*	Potluri et al., 2012
BW25113Δ6LDT	BW25113 Δ*ldtA,* Δ*ldtB,* Δ*ldtC,* Δ*ldtD,* Δ*ldtE,* Δ*ldtF*::*kan*	Kuru et al., 2017
BW25113Δ6LDTΔ*dacA*	BW25113 Δ*ldtA,* Δ*ldtB,* Δ*ldtC,* Δ*ldtD,* Δ*ldtE,* Δ*ldtF,* Δ*dacA::kan*	This work
LOBSTR-BL21(DE3)	F– *ompT hsdS*B(rB– mB–) *gal dcm* (DE3), carries genomically modified copies of *arnA* and *slyD*	Kerafast

Table S2. Oligonucleotides

Primer	Sequence 5' > 3'	Description	Used to make
AMS-GA7-F	TCTAGAGTCGACCTGCAGGC ATGCCCATGGTCTGTTTC	pACYC linearization fwd	pAMS03-05
AMS-GA7-R	CTGTGTGAAATTATTTCACA CAGGAAACAGACCATG	pACYC linearization rev	pAMS03-05
AMS-GA7k-F	GATGCGTCGTGTAAATATTC TTTGCGCATGCCTGCAGG	*ldtA* fragment fwd	pAMS03(ErfK)
AMS-GA7k-R	TCGACTCTAGATTAAAACAT CTGTCTTGAACATTTCACACAGGAA	*ldtA* fragment rev	pAMS03(ErfK)
AMS-GA7y-F	ACAGACCATGGATGAATATG AAATTGAAAACGCATGCCTGCAG	*ldtB* fragment fwd	pAMS04(YbiS)
AMS-GA7y-R	GTCGACTCTAGATTAATTCAGACGAACCGGCATCCCATTTCACACAGGA	*ldtB* fragment rev	pAMS04(YbiS)
AMS-GA7c_F	AACAGACCATGGATGATCAAAACGCGTTTTTCGCATGCCTGCAG	*ldtC* fragment fwd	pAMS05(YcfS)
AMS-GA7c_R	GTCGACTCTAGATTACAGCGTTTGTGGGCTCAC	*ldtC* fragment rev	pAMS05(YcfS)
nm182	GGAGGCCATGGGTCCTCGCTTGTTAACCAAACGCGG	PBP1c fragment with *Nco*I site	pNM039
nm183	GGAGGGAATTCTTGCATGACAAATTTCACTGTCGCGATTTGCCC	PBP1c fragment with *Eco*RI site	pNM039

Table S3. Plasmids

Plasmids	Relevant characteristics	Source or Reference
pJEH012(LdtD)	pACYC184 derivative; expresses *ldtD* under the IPTG-inducible *trc* promoter; Tet^R	(Hugonnet et al., 2016)
pAMS01(LdtE)	pACYC184 derivative; expresses *ldtE* under the IPTG-inducible trc promoter; Tet^R	(Morè et al., 2018)[a]
pAMS02(LdtF)	pACYC184 derivative; expresses *ldtF* under the IPTG-inducible *trc* promoter; Tet^R	(Morè et al., 2018)[a]
pAMS03(LdtA)	pACYC184 derivative; expresses *ldtA* under the IPTG-inducible *trc* promoter; Tet^R	This work
pAMS04(LdtB)	pACYC184 derivative; expresses *ldtB* under the IPTG-inducible *trc* promoter; Tet^R	This work
pAMS05(LdtC)	pACYC184 derivative; expresses *ldtC* under the IPTG-inducible *trc* promoter; Tet^R	This work
pSAV057	p*trc*99A derivative; contains weakened -35 promotor region (TTGACA-TTTACA); p15 origin; cat^R	(Alexeeva et al., 2010)
pGS121	pGZ119H derivative; expresses *ldtE* under the *tac* promoter; cat^R	(Morè et al., 2018)[a]
pGS124	pGZ119H derivative, expresses *ldtF* under the *tac* promoter; cat^R	(Morè et al., 2018)[a]
pWA001	p*trc*99A derivative; expresses the mCherry-PBP1a under the IPTG-inducible weakened -35 promotor region (TTGACA-TTTACA); pBR322 origin; Amp^R.	Banzhaf et al., 2012
pUM1Bα	p*tac* derivative; expresses the PBP1bα (M^1-N^{844}) gene under the IPTG-inducible promoter; Kan^R.	Meisel et al., 2003
pUM1Bα*	p*tac* derivative; expresses the PBP1bα (M^1-(S^{510}A)-N^{844}) gene under the IPTG-inducible promoter; Kan^R.	Meisel et al., 2003
pUM1BTG*α	p*tac* derivative; expresses the PBP1bα (M^1-(E^{233}A)-N^{844}) gene under the IPTG-inducible promoter; Kan^R.	Meisel et al., 2003
pUM1BTG*α*	p*tac* derivative; expresses the PBP1bα (M^1-(E^{233}A:S^{510}A)-N^{844}) gene under the IPTG-inducible promoter; Kan^R.	Meisel et al., 2003
pNM004	p*trc*99A derivative; expresses the OmpA gene under the IPTG-inducible weakened -35 promotor region (TTGACA-TTTACA); pBR322 origin; Amp^R.	Meiresonne et al., 2017
pNM039	p*trc*99A derivative; expresses mCherry-PBP1c under the IPTG-inducible weakened -35 promotor region (TTGACA-TTTACA); pBR322 origin; Amp^R.	This work
pNM009	p*trc*99A derivative; expresses the PBP5 gene under the IPTG-inducible weakened -35 promotor region (TTGACA-TTTACA); p15 origin; cat^R.	Meiresonne et al., 2017

pAM6a	p*trc*99A derivative; expresses the PBP6a gene under the IPTG-inducible weakened -35 promotor region (TTGACA-TTTACA); p15 origin; cat^R.	Meiresonne et al., 2017
pAM6b	p*trc*99A derivative; expresses the PBP6b gene under the IPTG-inducible weakened -35 promotor region (TTGACA-TTTACA); p15 origin; cat^R.	Meiresonne et al., 2017
pETMM82 *dsbC*-His6-*ldtD*	pETMM82 derivative; expresses *ldtD* fused at N-terminal with DsbC and a 6×His tag	Hugonnet et al., 2016

[a] Morè, N., Martorana, A. M., Biboy, J., Otten, C., Winkle, M., Montón Silva, A., Atkinson, L., Yau, H., Breukink, E., den Blaauwen, T., Vollmer, W., and Polissi, A. (2018). Peptidoglycan remodeling enables *E. coli* to survive severe outer membrane assembly defect. Nature Microbiol. Under review.

Table S4. Transformants for PG labelling with NADA

Strain	Plasmid(s)	Protein(s) produced
BW25113	pJEH12(LdtD)	LdtD
BW25113Δ*lpoA*	pJEH12(LdtD)	LdtD
BW25113Δ*mrcA*	pJEH12(LdtD)	LdtD
BW25113Δ*lpoB*	pJEH12(LdtD)	LdtD
BW25113Δ*CpoB*	pJEH12(LdtD)	LdtD
BW25113Δ*mrcB*	pJEH12(LdtD)	LdtD
	pWA001	PBP1a
	pUM1Bα	PBP1b
	pUM1Bα*	PBP1b TP*
	pUM1BTG*α	PBP1b GT*
	pUM1BTG* α*	PBP1b GT*TP*
	pNM039	PBP1c
	pJEH12(LdtD)+ pWA001	LdtD + PBP1a
	pJEH12(LdtD) + pUM1Bα	LdtD + PBP1b
	pJEH12(LdtD) + pUM1Bα*	LdtD + PBP1b TP*
	pJEH12(LdtD) + pUM1BGT*α	LdtD + PBP1b GT*
	pJEH12(LdtD) + pUM1BGT*α*	LdtD + PBP1b GT*TP*
	pJEH12(LdtD) + pNM039	LdtD + PBP1c
BW25113Δ*pbpC*	pJEH12(LdtD)	LdtD
BW25113Δ*dacA*	pJEH12(LdtD)	LdtD
BW25113Δ*dacC*	pJEH12(LdtD)	LdtD
BW25113Δ*dacD*	pJEH12(LdtD)	LdtD
BW25113Δ6LDT	pJEH12(LdtD)	LdtD
	pAMS01(LdtE)	LdtE
	pAMS02(LdtF)	LdtF
	pAMS03(LdtA)	LdtA
	pAMS04(LdtB)	LdtB
	pAMS05(LdtC)	LdtC
	pAMS02(LdtF) + pGS121	LdtF + LdtE
	pJEH12(LdtD) + pGS124	LdtD + LdtF
BW25113Δ6LDTΔdacA	pJEH12(LdtD)	LdtD
	pNM009	PBP5
	pAM6a	PBP6a
	pAM6b	PBP6b
	pJEH12(LdtD) + pNM009	LdtD + PBP5
	pJEH12(LdtD) + pAM6a	LdtD + PBP6A
	pJEH12(LdtD) + pAM6b	LdtD + PBP6B

Figure S1. LdtD incorporates HADA into muropeptides and during ongoing PG de novo synthesis.

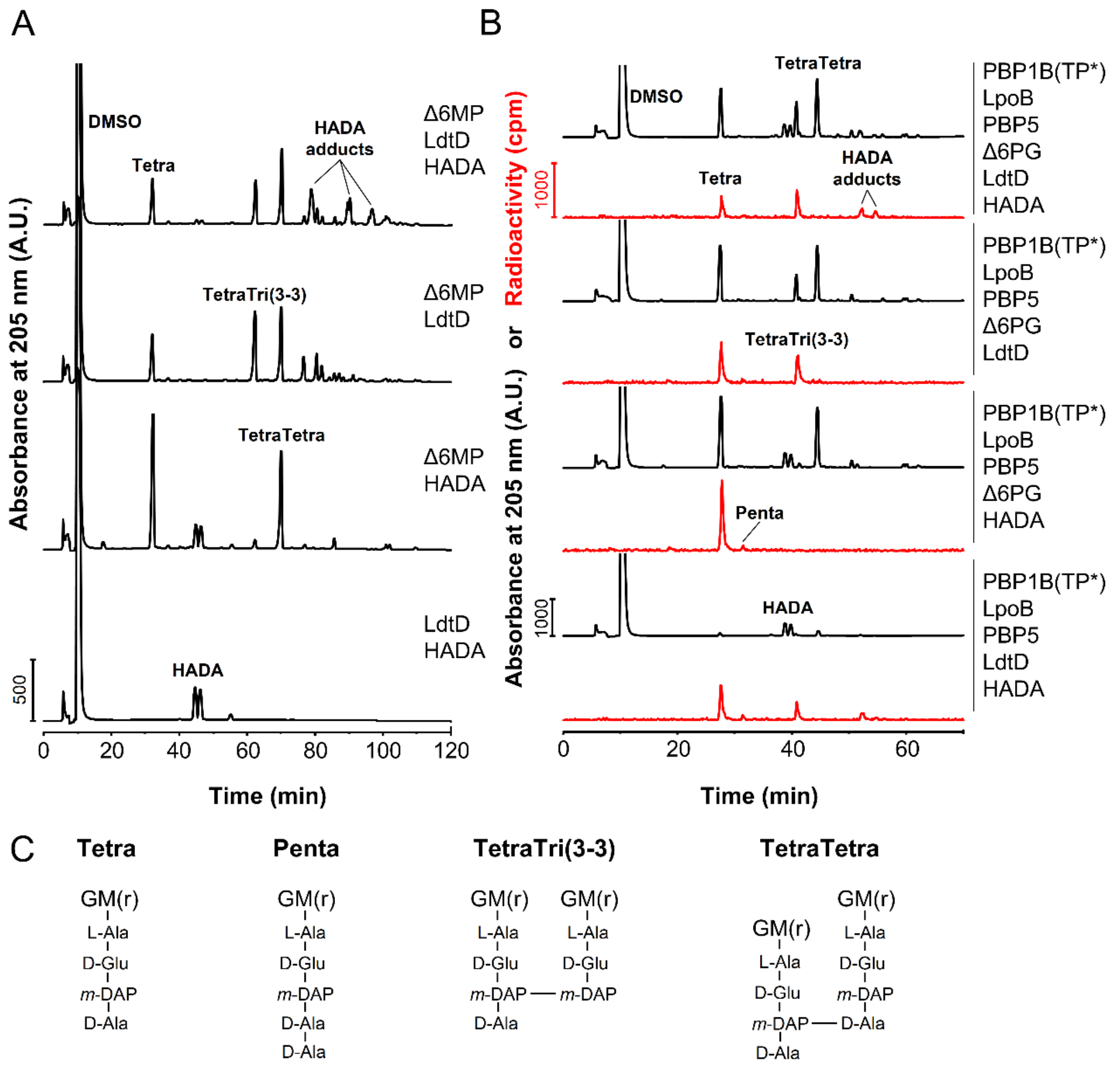

(A) HPLC chromatograms showing the formation of HADA adducts by LdtD incubated with muropeptides from BW25113Δ6LDT in the presence of HADA. Samples were reduced with sodium borohydride before HPLC analysis. **(B)** HPLC chromatograms obtained from samples upon incubating radioactive labeled lipid II, PG from BW25113Δ6LDT and the proteins indicated to the right in the presence of HADA. Samples were digested with cellosyl, reduced with sodium borohydride and subjected to HPLC analysis with detection of both UV signal (black traces) and radioactivity (red traces). PBP1b (TP*), PBP1b with an inactive transpeptidase site due to the replacement of Ser-510 by Ala. **(C)** Proposed structures of

muropeptides present in the fractions in panels A and B. G, N-acetylglucosamine; M, N-acetylmuramic acid; M(r), N-acetylmuramitol; M-P, N-acetylmuramic acid-1-phosphate; L-Ala, L-alanine; D-Glu, D-glutamic acid; D-Ala, D-alanine; m-DAP, meso-diaminopimelic acid.

Figure S2. Expressing *ldtD* improves incorporation of NADA

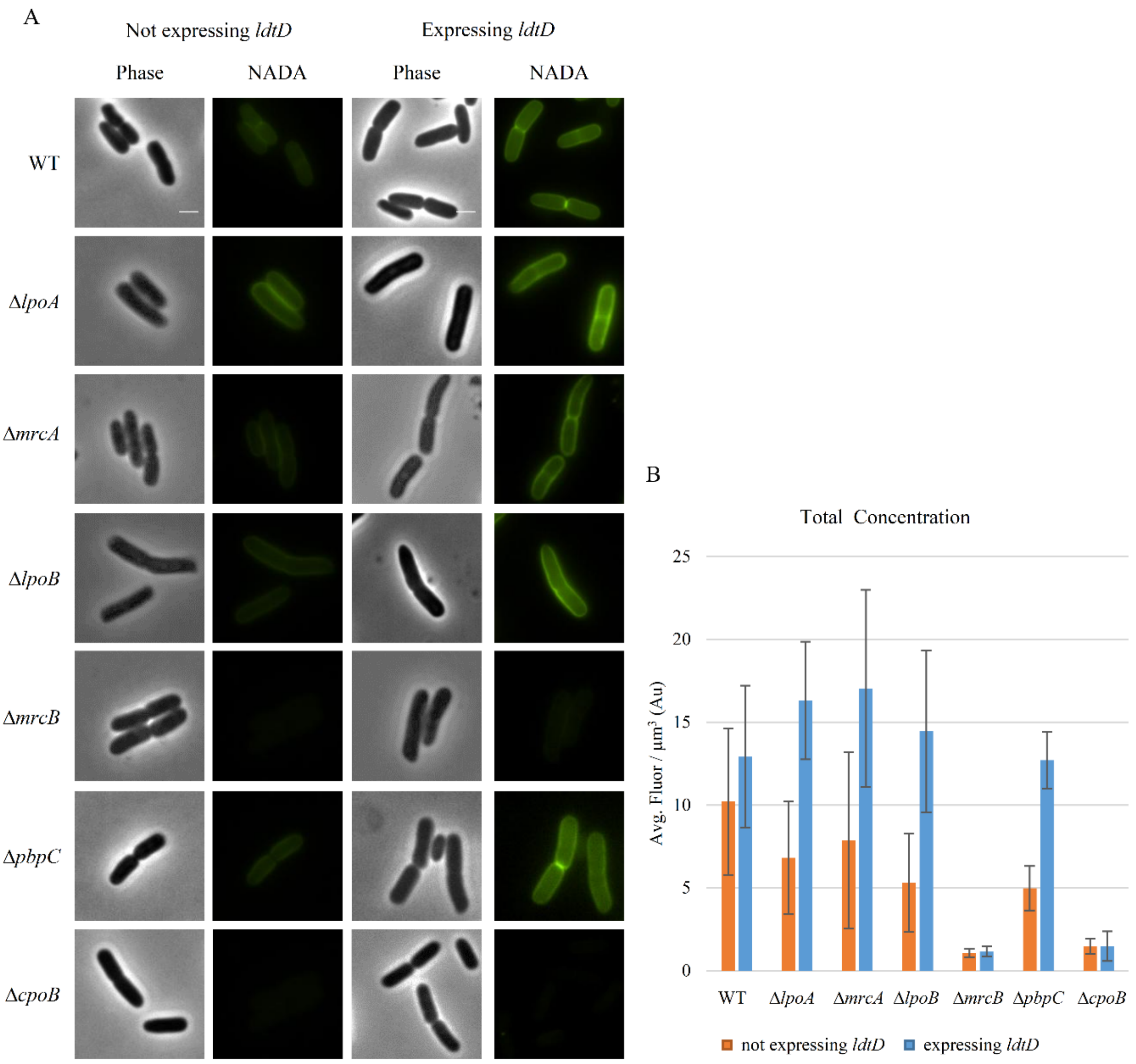

(A) Phase contrast and fluorescence images of empty cells (left panel) and *ldtD* expressing cells (right panel) in the strains (from top to bottom) WT BW25113, Δ*lpoA* (LpoA), Δ*mrcA* (PBP1a), Δ*lpoB* (LpoB), Δ*mrcB* (PBP1b), Δ*pbpC* (PBP1c) and Δ*cpoB* (CpoB). Cells were labelled with 0.5 mM NADA with a 2 min pulse. Scale bar represents 2 µm. **(B)** Total NADA concentration (signal per μm^3 average cell volume). Quantification of the incorporated NADA in the WT strain (in empty cells *n*=305; in *ldtD* expressing cells *n*=306), Δ*lpoA* (in empty cells *n*=905; in *ldtD* expressing cells *n*=469), Δ*mrcA* (in empty cells *n*=387; in *ldtD* expressing cells

n=402), Δ*lpoB* (in empty cells *n*=718; in LdtD expressing cells *n*=495), Δ*mrcB* (in empty cells *n*=286; in *ldtD* expressing cells *n*=453) Δ*pbpC* (in empty cells *n*=298; in *ldtD* expressing cells *n*=202) and Δ*cpoB* (in empty cells n=3804; in LdtD expressing cells *n*=1335). *n* is the number of cells analysed. Orange bars represent cells without plasmid for the expression of *ldtD* and blue bars represent cells expressing *ldtD*.

Figure S3. Cefsulodin-treated cells miss incorporation of NADA at mid-cell.

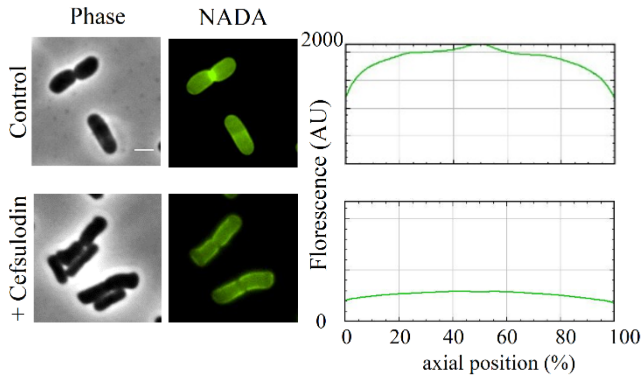

Phase contrast and corresponding fluorescence images of wild type BW25113 cells expressing *ldtD* without cefsulodin (control) or after 1 h incubation with 1 μg/mL cefsulodin in the presence of 0.5 mM NADA. Scale bar, 2 μm. Right panels show the average fluorescence profiles of the cells (from 0 to 2000 AU) plotted against normalized cell length (from 0 to 100%).

Figure S4. Overproduction of LdtD prevents cell lysis in BW25113Δ*mrcB* cells when the TPase activity of PBP1b is absent and a functional PBP1b GTase domain is present.

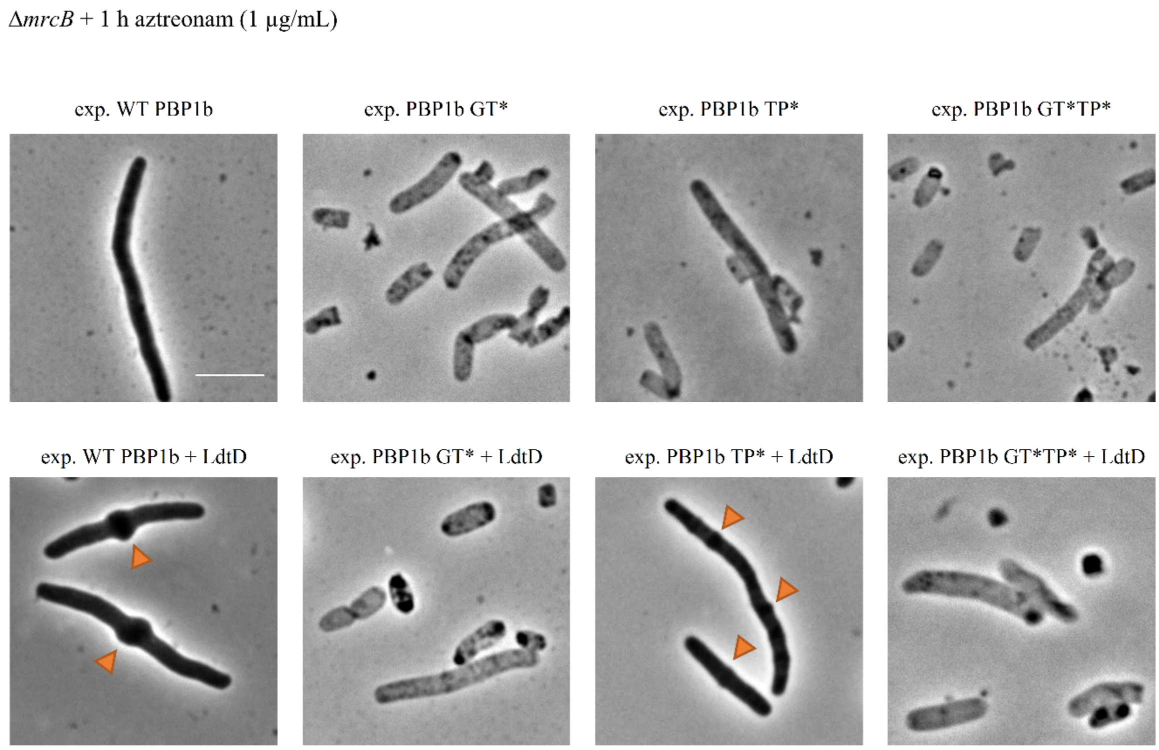

Phase contrast images of Δ*mrcB* cells producing, from left to right, PBP1b, PBP1b GT* (inactive GTase domain), PBP1b TP* (inactive TPase domain) and PBP1b GT*TP* (inactive GTase and TPase domains) alone (upper panel) or in combination with LdtD (lower panel) after 1 h treatment with 1 µg/mL aztreonam. Scale bar, 5 µm. Orange triangles point to the bulges at constriction sites.

References

1- Alexeeva, S., Gadella, T. W. J., Verheul, J., Verhoeven, G. S., & Blaauwen, den, T. (2010). Direct interactions of early and late assembling division proteins in Escherichia coli cells resolved by FRET. *Mol. Microbiol.* 77(2), 384–398. http://doi.org/10.1111/j.1365-2958.2010.07211.x

2- Baba, T., Ara, T., Hasegawa, M., Takai, Y., Okumura, Y., Baba, M., Datsenko, K. A., Tomita, M., Wanner, B. L., Mori, H. (2006). Construction of *Escherichia coli* K-12 in-frame, single-gene knockout mutants: the Keio collection. *Mol. Syst. Biol.* 2, 2006.0008. Epub 2006 Feb 21.

3- Datsenko, K. A., Wanner B.L. (2000). One-step inactivation of chromosomal genes in *Escherichia coli* K-12 using PCR products. *Proc. Natl. Acad. Sci.* 97, 6640–6645.

4- Denome, S. A., Elf, P. K., Henderson, T. A., Nelson, D. E., Young, K. D. (1999). *Escherichia coli* mutants lacking all possible combinations of eight penicillin binding proteins: Viability, characteristics, and implications for peptidoglycan synthesis. *J. Bacteriol.* 181, 3981–3993.

5- Gray, A. N., Egan, A. J. F., van't Veer, I. L., Verheul, J., Colavin, A., Koumoutsi, A., Biboy, J., Altelaar, A. F. M., Damen, M. J., Huang, K. C., Simorre, J. P., Breukink, E., den Blaauwen, T., Typas, A., Gross, C. A., and Vollmer, W. (2015). Coordination of peptidoglycan synthesis and outer membrane constriction during *Escherichia coli* cell division. *eLife* 4, e07118.

6- Hugonnet, J. E., Mengin-Lecreulx, D., Monton, A., den Blaauwen, T., Carbonnelle, E., Veckerlé, C., Brun, Y. V., van Nieuwenhze, M., Bouchier, C., Tu, K., Rice, L. B., and Arthur, M. (2016). Factors essential for L,D-transpeptidase mediated peptidoglycan cross-linking and β-lactam resistance in *Escherichia coli*. *eLife* 5. e19469. doi: 10.7554/eLife.19469.

7- Kuru, E., Hughes, V. H., Brown, P. J., Hall, E., Tekkam, S., Cava, F., de Pedro, M. A., Brun, Y. V., and van Nieuwenhze, M. S. (2012). In Situ Probing of Newly Synthesized Peptidoglycan in Live Bacteria with Fluorescent d-Amino Acids. *Angew. Chem. Int. Ed.* 51, 12519 –12523.

8- Kuru, K., Lambert, C., Rittichier, J., Till, R., Ducret, A., Derouaux, A., Gray, J., Biboy, J., Vollmer, W., VanNieuwenhze, M., Brun, Y. V., Sockett, R. E. (2017). Fluorescent D-amino-acids reveal bi-cellular cell wall modifications important for *Bdellovibrio bacteriovorus* predation. *Nat. Microbiol.* 2(12), 1648-1657. doi: 10.1038/s41564-017-0029-y.

9- Meiresonne, N. Y., van der Ploeg, R., Hink, M. A., and den Blaauwen T. (2017). Activity-Related Conformational Changes in d,d-Carboxypeptidases Revealed by In Vivo Periplasmic Förster Resonance Energy Transfer Assay in *Escherichia coli*. *mBio* 8(5), e01089-17. doi: 10.1128/mBio.01089-17.

10- Meisel, U., Hoeltje, J. V., Vollmer, W. (2003). Overproduction of inactive variants of the murein synthase PBP1B causes lysis in *Escherichia coli*. *J Bacteriol.* 185(18), 5342–5348.

11- Morè, N., Martorana, A. M., Biboy, J., Otten, C., Winkle, M., Montón Silva, A., Atkinson, L., Yau, H., Breukink, E., den Blaauwen, T., Vollmer, W., and Polissi, A. (2018). Peptidoglycan remodeling enables *E. coli* to survive severe outer membrane assembly defect. Manuscript submitted.

12- Potluri, L. P., de Pedro, M. A., Young, K. D. (2012). *Escherichia coli* low-molecular-weight penicillin-binding proteins help orient septal FtsZ, and their absence leads to asymmetric cell division and branching. *Mol. Microbiol.* 84(2), 203-24.
